# Feature Selection and Cancer Classification via Sparse Logistic Regression with the Hybrid L_1/2 +2_ Regularization

**DOI:** 10.1371/journal.pone.0149675

**Published:** 2016-05-02

**Authors:** Hai-Hui Huang, Xiao-Ying Liu, Yong Liang

**Affiliations:** Faculty of Information Technology & State Key Laboratory of Quality Research in Chinese Medicines, Macau University of Science and Technology, Avenida Wai Long, Taipa, Macau, 999078, China; Jilin University, CHINA

## Abstract

Cancer classification and feature (gene) selection plays an important role in knowledge discovery in genomic data. Although logistic regression is one of the most popular classification methods, it does not induce feature selection. In this paper, we presented a new hybrid L_1/2 +2_ regularization (HLR) function, a linear combination of L_1/2_ and L_2_ penalties, to select the relevant gene in the logistic regression. The HLR approach inherits some fascinating characteristics from L_1/2_ (sparsity) and L_2_ (grouping effect where highly correlated variables are in or out a model together) penalties. We also proposed a novel univariate HLR thresholding approach to update the estimated coefficients and developed the coordinate descent algorithm for the HLR penalized logistic regression model. The empirical results and simulations indicate that the proposed method is highly competitive amongst several state-of-the-art methods.

## 1. Introduction

With advances in high-throughput molecular techniques, the researchers can study the expression of tens of thousands of genes simultaneously. Cancer classification based on gene expression levels is one of the central problems in genome research. Logistic regression is a popular classification method and has an explicit statistical interpretation which can obtain probabilities of classification regarding the cancer phenotype. However, in most gene expression studies, the number of genes typically far exceeds the number of the sample size. This situation is called high-dimensional and low sample size problem, and the normal logistic regression method cannot be directly used to estimate the regression parameters.

To deal with the problem of high dimensionality, one of the popular techniques is the regularization method. A well-known regularization method is the L_1_ penalty [1], which is the least absolute shrinkage and selection operator (Lasso). It is performing continuous shrinkage and gene selection at the same time. Other L_1_ norm type regularization methods typically include the smoothly-clipped-absolute-deviation (SCAD) penalty [2], which is symmetric, nonconcave, and has singularities at the origin to produce sparse solutions. The adaptive Lasso [3] penalizes the different coefficients with the dynamic weights in the L_1_ penalty. However, the L_1_ type regularization may yield inconsistent feature selections in some situations [3] and often introduces extra bias in the estimation of the parameters in the logistic regression [4]. Xu *et al*. [5] proposed the L_1/2_ penalty, a method that can be taken as a representative of L_q_ (0 <*q* < 1) penalties in both sparsity and computational efficiency, and has demonstrated many attractive properties, such as unbiasedness, and oracle properties [5–7]. However, similar to most of the regularization methods, the L_1/2_ penalty ignores the correlation between features, and consequently unable to analyze data with dependent structures. If there is a group of variables among which the pair-wise correlations are very high, then the L_1/2_ method tends to select only one variable to represents the corresponding group. In gene expression study, genes are often highly correlated if they share the same biological pathway [8]. Some efforts had been made to deal with the problem of highly correlated variables. Zhou and Hastie proposed Elastic net penalty [9] which is a linear combination of L_1_ and L_2_ (the ridge technique) penalties, and such method emphasizes a grouping effect, where strongly correlated genes tend to be in or out of the model together. Becker *et al*. [10] proposed the Elastic SCAD (SCAD − L_2_), a combination of SCAD and L_2_ penalties. By introducing the L_2_ penalty term, Elastic SCAD also works for the groups of predictors.

In this article, we proposed the HLR (Hybrid L_1/2 + 2_ Regularization) approach to fit the logistic regression models for gene selection, where the regularization is a linear combination of the L_1/2_ and L_2_ penalties. The L_1/2_ penalty achieves feature selection. In theory, a strictly convex penalty function provides a sufficient condition for the grouping effect of variables and the L_2_ penalty guarantees strict convexity [11]. Therefore, the L_2_ penalty induces the grouping effect simultaneously in the HLR approach. Experimental results on artificial and real gene expression data in this paper demonstrate that our proposed method is very promising.

The rest of the article is organized as follows. In Section 2, we first defined the HLR approach and presented an efficient algorithm for solving the logistic regression model with the HLR penalty. In Section 3, we evaluated the performance of our proposed approach on the simulated data and five public gene expression datasets. We presented a conclusion of the paper in Section 4.

## 2. Methods

### 2.1 Regularization

Suppose that dataset *D* has *n* samples *D* = {(*X*_1_, *y*_1_), (*X*_2_, *y*_2_),…,(*X*_*n*_, *y*_*n*_)}, where *X*_*i*_ = (*x*_*i*1_, *x*_*i*2_, …, *x*_*ip*_) is *i*^th^ sample with *p* dimensional and *y*_*i*_ is the corresponding dependent variable.

For any non-negative *λ*, the normal regularization form is:

$$L\left(\lambda ,\beta \right)=\text{argmin}\frac{1}{n}\sum_{i=1}^{n}{\left(y-X^{\prime }\beta \right)}^{2}+\lambda P\left(\beta \right)$$ (1)

where *P*(*β*) represents the regularization term. There are many regularization methods proposed in recent years. One of the popular methods is the L_1_ regularization (Lasso), where $P\left(\beta \right)=\sum_{j=1}^{p}{\left|\beta_{j}\right|}^{1}$. The others L_1_ type regularizations include SCAD, the adaptive Lasso, Elastic net, Stage wise Lasso [12], Dantzig selector [13] and Elastic SCAD. However, in genomic research, the result of the L_1_ type regularization may not sparse enough for interpretation. Actually, a typical microarray or RNA-seq data set has many thousands of predictors (genes), and researchers often desire to select fewer but informative genes. Beside this, the L_1_ regularization is asymptotically biased [14,15]. Although the L_0_ regularization, where $P\left(\beta \right)=\sum_{j=1}^{p}{\left|\beta_{j}\right|}^{0}$, yields the sparsest solutions, it has to deal with NP-hard combinatory optimization problem. To gain a more concise solution and improve the predictive accuracy of the classification model, we need to think beyond the L_1_ and L_0_ regularizations to the L_q_ (0<*q*<1) regularization. The L_1/2_ regularization can be taken as a representative of the L_q_ (0<*q*<1) penalties and has permitted an analytically expressive thresholding representation [5]. With the thresholding representation, solving the L_1/2_ regularization is much easier than solving the L_0_ regularization. Moreover, the L_1/2_ penalty is unbiasedness and has oracle properties [5–7]. These characteristics are making the L_1/2_ penalty became an efficient tool for high dimensional problems [16,17]. However, due to the insensitivity of the highly correlated data, the L_1/2_ penalty tends to select only one variable to represent the correlated group. This drawback may deteriorate the performance of the L_1/2_ method.

### 2.2 Hybrid L_1/2 +2_ Regularization (HLR)

For any fixed non-negative λ_1_ and λ_2_, we define the hybrid L_1/2 +2_ regularization (HLR) criterion:

$$L\left(\lambda_{1},\lambda_{2},\beta \right)=\text{argmin}\frac{1}{n}\sum_{i=1}^{n}{\left(y-X^{\prime }\beta \right)}^{2}+\lambda_{1}{\left|\beta \right|}_{1/2}+\lambda_{2}{\left|\beta \right|}^{2}$$ (2)

where *β* = (*β*_1_, …, *β*_*p*_) are the coefficients to be estimated and

$${\left|\beta \right|}_{1/2}=\sum_{j=1}^{p}{\left|\beta_{j}\right|}^{1/2},$$

$${\left|\beta \right|}^{2}=\sum_{j=1}^{p}{\left|\beta_{j}\right|}^{2}.$$

The HLR estimator $\hat{\beta }$ is the minimizer of Eq (2):

$$\hat{\beta }={\text{argmin}}_{\beta }\left\{L\left(\lambda_{1},\lambda_{2},\beta \right)\right\}.$$ (3)

Let α = *λ*_1_/(1 + *λ*_2_), then solving $\hat{\beta }$ in Eq (3) is equivalent to the optimization problem:

$$\hat{\beta }={\text{argmin}}_{\beta }\{{\left|\;\text{y}-{\text{X}}^{\prime }\beta \right|}^{2}+\lambda \left(\alpha {\left|\beta \right|}_{1/2}+\left(1-\alpha \right){\left|\beta \right|}^{2}\right)\}$$ (4)

We call the function *α*|*β*|_1/2_ + (1 − α)|*β*|^2^ as the HLR, which is a combination of the L_1/2_ and L_2_ penalties. When α = 0, the HLR penalty becomes ridge regularization. When α = 1, the HLR becomes L_1/2_ regularization. The L_2_ penalty is enjoying the grouping effect and the L_1/2_ penalty induces sparse solutions. This combination of the both penalties makes the HLR approach not only capable of dealing with the correlation data, but also able to generate a succinct result.

**Fig 1:**
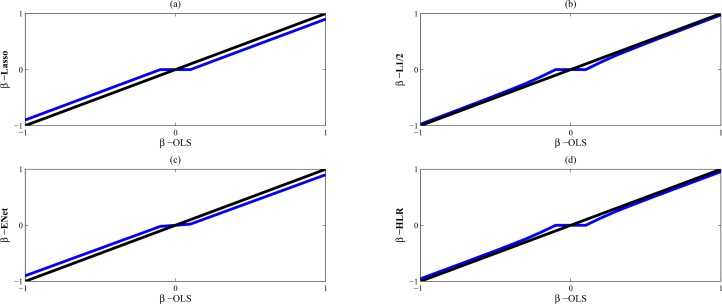
**Exact solutions of (a) Lasso, (b) L_1/2_, (c) Elastic net, and (d) HLR in an orthogonal design.** The regularization parameters are *λ* = 0.1 and *α* = 0.8 for Elastic net and HLR. *(β-OLS is the ordinary least-squares (OLS) estimator)*.

Fig 1 shows four regularization methods: Lasso, L_1/2_, Elastic net and HLR penalties with an orthogonal design matrix in the regression model. The estimators of Lasso and Elastic net are biased, whereas the L_1/2_ penalty is asymptotically unbiased. Similar to the L_1/2_ method, the HLR approach also performs better than Lasso and Elastic net in the property of unbiasedness.

**Fig 2:**
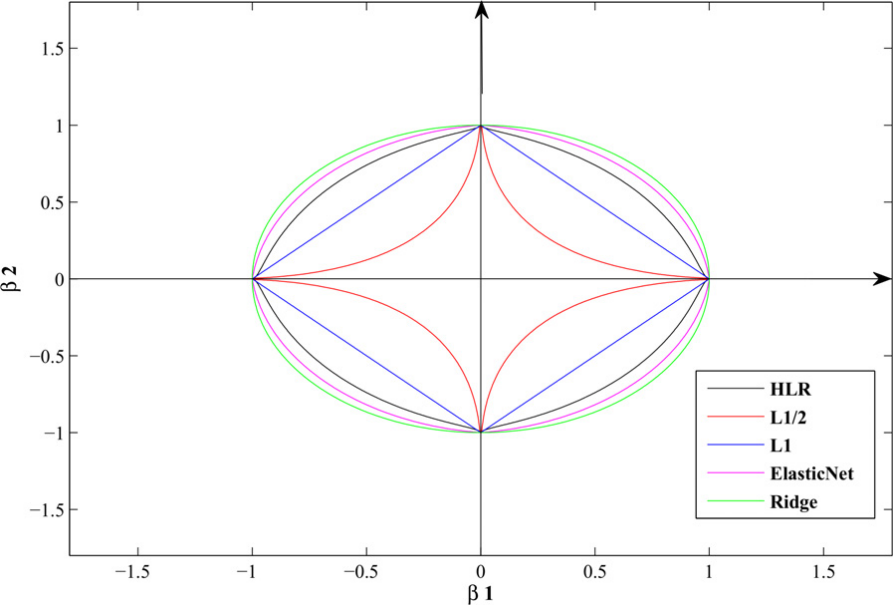
Contour plots (two-dimensional) for the regularization methods. The regularization parameters are *λ* = 1 and *α* = 0.2 for the HLR method.

Fig 2 describes the contour plots on two-dimensional for the penalty functions of Lasso, Elastic net, L_1/2_ and HLR approaches. It is suggest that the L_1/2_ penalty is non-convex, whereas the HLR is convex for the given α. The following theorem will show how the HLR strengthens the L_1/2_ regularization.

#### Theorem 1

Given dataset (y, X) and (λ_1_, λ_2_), then the HLR estimates $\hat{\beta }$ are given by

$$\hat{\beta }={\text{argmin}}_{\beta }\beta^{T}\left(\frac{X^{T}X\;+\;\lambda_{2}I}{1+\;\lambda_{2}}\right)\beta -2y^{T}X\beta +\;\lambda_{1}{\left|\beta \right|}^{1/2}\;.$$ (5)

The L_1/2_ regularization can be rewritten as

$$\hat{\beta }\left({\text{L}}_{1/2}\right)={\text{argmin}}_{\beta }\beta^{T}\left(X^{T}X\right)\beta -2y^{T}X\beta +\;\lambda_{1}{\left|\beta \right|}^{1/2}\;.$$ (6)

The proof of Theorem 1 can be found in S1 File. Therorem1 shows the HLR approach is a stabilized version of the L_1/2_ regularization. Note that $\hat{\sum }=X^{T}X$ is a sample version of the correlation matrix Σ and

$$\frac{X^{T}X+\lambda_{2}I}{1+\;\lambda_{2}}=\left(1-\;\delta \right)\hat{\sum }+\;\delta I,$$

where *δ* = *λ*_2_/(1 + *λ*_2_) shrinks $\hat{\sum }$ that towards the identity matrix. The classification accuracy can often be enhanced by replacing $\hat{\sum }$ by a more shrunken estimate in linear discriminate analysis [18,19]. In other word, the HLR improves the L_1/2_ technique by regularizing $\hat{\sum }$ in Eq (6).

### 2.3 The sparse logistic regression with the HLR method

Suppose that dataset *D* has *n* samples *D* = {(*X*_1_, *y*_1_), (*X*_2_, *y*_2_), …, (*X*_*n*_, *y*_*n*_)}, where *X*_*i*_ = (*x*_*i*1_, *x*_*i*2_, …, *x*_*ip*_) is *i*^th^ sample with *p* genes and *y*_*i*_ is the corresponding dependent variable that consist of a binary value with 0 or 1. Define a classifier f(*x*) = *e*^*x*^ / (1 + *e*^*x*^) and the logistic regression is defined as:

$$P(y_{i}\;=\;1|X_{i})\;=\;\text{f}\left(X\prime_{i}\beta \right)\;=\;\frac{exp\left({X^{\prime }}_{i}\beta \right)}{1+exp\left({X^{\prime }}_{i}\beta \right)}$$ (7)

Where *β* = (*β*_1_, …, *β*_*p*_) are the coefficients to be estimated. With a simple algebra, the regression model can be presented as:

$$L\left(\beta \right)=-\sum_{i=1}^{n}\left\{\;y_{i}log\left[\text{f}\left(X\prime_{i}\beta \right)\right]+\left(1-y_{i}\right)\text{log}\left[1-\text{f}\left(X\prime_{i}\beta \right)\right]\;\right\}$$ (8)

In this paper, we apply the HLR approach to the logistic regression model. For any fixed non-negative *λ* and *α*, the sparse logistic regression model based on the HLR approach is defined as:

$$L\left(\lambda ,\;\alpha ,\;\beta \right)=-\sum_{i=1}^{n}\left\{\;y_{i}\;\text{log}\left[\text{f}\left({X^{\prime }}_{i}\beta \right)\right]+\left(1-y_{i}\right)\;\text{log}\left[1-\text{f}\left({X^{\prime }}_{i}\beta \right)\right]\right\}+\lambda \left(\alpha {\left|\beta \right|}_{1/2}+\left(1-\alpha \right){\left|\beta \right|}^{2}\right)$$ (9)

### 2.4 Solving algorithm for the sparse logistic regression with the HLR approach

The coordinate descent algorithm [20] is an efficient method for solving regularization models because its computational time increases linearly with the dimension of the problems. Its standard procedure can be showed as follows: for every *β*_j_ (j = 1,2,…,*p*), to partially optimize the target function with respect to coefficient with the remaining elements of *β* fixed at their most recently updated values, iteratively cycling through all coefficients until meet converged. The specific form of renewing coefficients is associated with the thresholding operator of the penalty.

Suppose that dataset *D* has *n* samples *D* = {(*X*_1_, *y*_1_), (*X*_2_, *y*_2_), …, (*X*_*n*_, *y*_*n*_)}, where *X*_*i*_ = (*x*_*i*1_, *x*_*i*2_, …, *x*_*ip*_) is *i*^th^ sample with *p* dimensional and *y*_*i*_ is the corresponding dependent variable. The variables are standardized: $\sum_{i=1}^{n}\;x_{ij}^{2}=1$.

Following Friedman *et al*. [20] and Liang *et al*. [16], in this paper, we present the original coordinate-wise update form for the HLR approach:

$$\beta_{j}\leftarrow \;\frac{Half\left(\omega_{j},\lambda \alpha \right)}{1+\lambda \left(1-\alpha \right)}$$ (10)

where $\omega_{j}=\sum_{i=1}^{n}x_{ij}\left(y_{i}-{\tilde{y}}_{i}{}^{\left(j\right)}\right)$, and ${\tilde{y}}_{i}{}^{\left(j\right)}=\sum_{k\neq j}x_{ik}\beta_{k}$ as the partial residual for fitting *β*_*j*_. Half(z,r) is the L_1/2_ thresholding operator

$$Half\left(\omega_{j},\lambda \right)=\{\begin{array}{l} \frac{2}{3}\omega_{j}\left(1+\cos\left(\frac{2\left(\pi -\;\varphi_{\lambda }\left(\omega_{j}\right)\right)}{3}\right)\right)\;if\;\left|\omega_{j}\right|>\frac{3}{4}{\left(\lambda \right)}^{\frac{2}{3}}\\ \;0\;\text{otherwise} \end{array}$$ (11)

where $\varphi_{\lambda }\left(\omega \right)=arccos(\frac{\lambda }{8}{\left(\frac{\left|\omega \right|}{3}\right)}^{-\frac{3}{2}})$, *π* = 3.14.

The Eq (9) can be linearized by one-term Taylor series expansion:

$$L\left(\lambda ,\;\alpha ,\;\beta \right)\approx \frac{1}{2n}\sum_{i=1}^{n}{\left(Z_{i}-X_{i}\beta \right)}^{\prime }W_{i}\left(Z_{i}-X_{i}\beta \right)+\lambda \left(\alpha {\left|\beta \right|}_{1/2}+\left(1-\alpha \right){\left|\beta \right|}^{2}\right)$$ (12)

where $Z_{i}=X_{i}\tilde{\beta }+\frac{y_{i}-f\left(X_{i}\tilde{\beta }\right)}{f\left(X_{i}\tilde{\beta }\right)(1-f\left(X_{i}\tilde{\beta }\right)}$ is the estimated response, $W_{i}\;=\;f\left(X_{i}\tilde{\beta }\right)(1-f\left(X_{i}\tilde{\beta }\right)$ is the weight for the estimated response. $f\left(X_{i}\tilde{\beta }\right)\;=\;\text{exp}\left(X_{i}\tilde{\beta }\right)/\left(1+\text{exp}\left(X_{i}\tilde{\beta }\right)\right)$ is the evaluated value under the current parameters. Thus, we can redefine the partial residual for fitting current $\tilde{\beta }$ as ${\overset{ˇ}{Z}}_{i}^{\left(j\right)}\;=\;\sum_{k\neq j}x_{ik}{\tilde{\beta }}_{k}$ and $\omega_{j}=\sum_{i=1}^{n}W_{i}x_{ij}(Z_{i}-{\overset{ˇ}{Z}}_{i}^{\left(j\right)})$. The procedure of the coordinate descent algorithm for the HLR penalized logistic model is described as follows.

### Algorithm: The coordinate descent approach for the HLR penalized logistic model

Step 1: Initialize all *β*_*j*_(*m*) ← 0 (*j* = 1, 2,…,*p*) and *X*, *y*,
set *m* ← 0, *λ* and *α* are chosen by cross-validation;
Step 2: Calculate *Z*(*m*) and *W*(*m*) and approximate the loss function (12) based on the current *β*(*m*);
Step 3: Update each *β*_*j*_(*m*), and cycle over *j =* 1,…, *p*;
Step 3.1: Compute ${\overset{ˇ}{Z}}_{i}{}^{\left(j\right)}\left(m\right)\leftarrow \sum_{k\neq j}x_{ik}\beta_{k}\left(m\right)$ and $\omega_{j}\left(m\right)\leftarrow \sum_{i=1}^{n}W_{i}\left(m\right)x_{ij}\left(Z_{i}\left(m\right)-{\overset{ˇ}{Z}}_{i}{}^{\left(j\right)}\left(m\right)\right)$;
Step 3.2: Update $\beta_{j}\left(m\right)\leftarrow \frac{Half\left(\omega_{j}\left(m\right),\;\lambda \alpha \right)}{1+\lambda \left(1-\alpha \right)};$
Step 4: Let *m* ← *m* + 1, *β*(*m* + 1) ← *β*(*m*);
If *β*(*m*) dose not convergence, then repeat Steps 2, 3;

## 3. Results and Discussion

### 3.1 Analyzes of simulated data

The goal of this section is to evaluate the performance of the logistic regression with the HLR approach in the simulation study. Four approaches are compared with our proposed method: logistic regression with the Lasso regularization, L_1/2_ regularization, SCAD − L_2_ and Elastic net regularization respectively. We simulate data from the true model

$$\text{log}\left(\frac{y}{1-y}\right)=X\beta \;+\sigma \epsilon ,\;\epsilon ∼\;N\left(0,\;1\right),$$

where X ∼ *N*(0, 1), *ϵ* is the independent random error and *σ* is the parameter that controls the signal to noise. Four scenarios are presented here. In every example, the dimension of predictors is 1000. The notation. /. was represented the number of observations in the training and test sets respectively, e.g. 100/100. Here are the details of the four scenarios.

1. In scenario 1, the dataset consists of 100/100 observations, we set *σ* = 0.3 and
  β=(2,2,2,2,2,⏟50,…,0⏟995),
  we simulated a grouped variable situation
  $$x_{i}=\rho \times x_{1}+\left(1-\rho \right)\times x_{i},\text{i}=2,3,4,5;$$
  where *ρ* is the correlation coefficient of the grouped variables.
2. The scenario 2 was defined similarly to the scenario 1, except that we considered the case when there are other independent factors also contributes to the corresponding classification variable *y*,
  β=(2,2,2,2,2,1.5,−2,1.7,3,−2.5,⏟100,…,0⏟990).
3. In scenario 3, we set *σ* = 0.4 and the dataset consist of 200/200 observations, and
  β=(2,2,2,2,2,1.5,−2,1.7,3,−2.5,⏟103,…,3,⏟200,…,0⏟970),
  we defined two grouped variables
  $$x_{i}=\rho \times x_{1}+\left(1-\rho \right)\times x_{i},\text{i}=2,3,4,5;$$
  $$x_{i}=\rho \times x_{11}+\left(1-\rho \right)\times x_{i},\text{i}=12,\ldots \;,30;$$
4. In scenario 4, the true features were added up to 20% of the total features, *σ* = 0.4 and the dataset consist of 400/400 observations, and
  β=(3,…,3,⏟30−2.5,2,−1.5,1.8,−2.5,⏟53,…,3,⏟402,…,2,⏟253,…,3,⏟302,…,2,⏟700,…,0⏟800),
  we defined three grouped variables
  $$x_{i}=\rho \times x_{1}+\left(1-\rho \right)\times x_{i},\text{i}=2,\ldots \;,30;$$
  $$x_{i}=\rho \times x_{36}+\left(1-\rho \right)\times x_{i},\text{i}=37,\ldots \;,75;$$
  $$x_{i}=\rho \times x_{101}+\left(1-\rho \right)\times x_{i},\text{i}=102,\ldots \;,130;$$

In this example, there were three groups of the correlated features and some single independent features. An ideal sparse regression method would select only the 200 true features and set the coefficients of the 800 noise features to zero.

In our experiment, we set the correlation coefficient *ρ* of features are 0.3, 0.6, 0.9 respectively. The Lasso and Elastic net were conducted by Glmnet (a Matlab package, version 2014-04-28, download at http://web.stanford.edu/~hastie/glmnet_matlab/). The optimal regularization parameters or tuning parameters (balance the tradeoff between data fit and model complexity) of the Lasso, L_1/2_, SCAD − L_2_, Elastic net and the HLR approaches were tuned by the 10-fold cross-validation (CV) approach in the training set. Note that, the Elastic net and HLR methods were tuned by the 10-CV approach on the two-dimensional parameter surfaces. The SCAD − L_2_ were tuned by the 10-CV approach on the three-dimensional parameter surfaces. Then, the different classifiers were built by these sparse logistic regressions with the estimated tuning parameters. Finally, the obtained classifiers were applied to the test set for classification and prediction.

We repeated the simulations 500 times for each penalty method and computed the mean classification accuracy on the test sets. To evaluate the quality of the selected features for the regularization approaches, the sensitivity and specificity of the feature selection performance [21] were defined as the follows:

$$\text{True Negative}\;\left(\text{TN}\right)≔{\left|\bar{\beta }.*\bar{\hat{\beta }}\right|}_{0},\;\text{False Positive}\;\left(\text{FP}\right)≔{\left|\bar{\beta }.*\hat{\beta }\right|}_{0}$$

$$\text{False Negative}\;\left(\text{FN}\right)≔{\left|\beta .*\bar{\hat{\beta }}\right|}_{0},\;\text{True Positive}\;\left(\text{TP}\right)≔{\left|\beta .*\hat{\beta }\right|}_{0}$$

$$\text{Sensitivity}≔\frac{\text{TP}}{\text{TP}+\text{FN}}\;,\;\text{Specificity}≔\frac{\text{TN}}{\text{TN}+\text{FP}}\;.$$

where the .* is the element-wise product, and |.|_0_ calculates the number of non-zero elements in a vector, $\bar{\beta }$ and $\bar{\hat{\beta }}$ are the logical “not” operators on the vectors *β* and $\hat{\beta }$.

As showed in Table 1, for all scenarios, our proposed HLR procedure generally gave higher or comparable classification accuracy than the Lasso, SCAD − L_2_, Elastic net and L_1/2_ methods. Also, the HLR approach results in much higher sensitivity for identifying true features compared to the other four algorithms. For example, in the scenario 1 with *ρ* = 0.9, our proposed method gained the impressive performance (accuracy 99.87% with perfect sensitivity and specificity). The specificity of the HLR approach is somewhat decreased, but not greatly as compared to the achieved in sensitivity.

**Table 1:** Mean results of the simulation. In bold–the best performance amongst all the methods.

		Scenario											
*ρ*	Method	1	2	3	4	1	2	3	4	1	2	3	4
		Sensitivity of feature selection				Specificity of feature selection				Accuracy of classification (test set)			
	**Lasso**	0.966	0.798	0.344	0.361	0.996	0.968	0.967	0.966	89.26%	81.47%	84.76%	80.26%
	**L**_**1/2**_	0.971	0.888	0.411	0.355	0.998	**0.974**	**0.975**	**0.970**	92.05%	82.22%	**85.11%**	81.45%
0.3	**SCAD − L**_**2**_	1.000	0.913	0.722	0.674	0.995	0.928	0.890	0.723	93.21%	**82.90%**	84.51%	82.51%
	**EN**	0.997	0.916	0.737	0.662	0.994	0.926	0.886	0.735	91.03%	81.34%	84.47%	80.27%
	**HLR**	1.000	**0.924**	**0.791**	**0.708**	**0.999**	0.931	0.892	0.769	**95.27%**	82.66%	84.99%	85.05%
	**Lasso**	0.887	0.723	0.351	0.270	0.995	**0.975**	0.981	0.923	94.24%	84.10%	91.88%	85.88%
	**L**_**1/2**_	0.755	0.630	0.275	0.220	1.000	0.974	**0.988**	**0.928**	95.90%	86.50%	90.20%	84.20%
0.6	**SCAD − L**_**2**_	1.000	0.866	0.800	0.629	1.000	0.949	0.929	0.849	96.33%	86.43%	89.20%	**93.03%**
	**EN**	1.000	0.854	0.795	0.621	1.000	0.953	0.939	0.837	96.22%	86.41%	92.12%	91.01%
	**HLR**	1.000	**0.875**	**0.816**	**0.636**	1.000	0.968	0.942	0.841	**99.53%**	**87.16%**	**92.71%**	92.82%
	**Lasso**	0.548	0.548	0.174	0.145	0.938	0.972	0.987	0.934	96.05%	86.79%	93.22%	91.15%
	**L**_**1/2**_	0.337	0.495	0.159	0.139	0.999	**0.977**	**0.991**	**0.944**	97.89%	87.90%	93.70%	92.70%
0.9	**SCAD − L**_**2**_	1.000	0.872	0.809	0.636	1.000	0.954	0.952	0.861	97.28%	88.60%	93.70%	93.19%
	**EN**	1.000	0.856	0.818	0.622	0.995	0.951	0.949	0.875	98.22%	88.14%	93.52%	93.82%
	**HLR**	1.000	**0.897**	**0.824**	**0.645**	1.000	0.966	0.956	0.880	**99.87%**	**89.40%**	**94.76%**	**94.40%**

*Mean results are based on 500 repeats*. *The sensitivity and specificity are both dedicated to measur*es *the quality of the selected features*, *the accuracy evaluates the classification performance of the different regularization approaches on the test sets*.

### 3.2 Analyzes of real data

To further evaluate the effectiveness of our proposed method, in this section, we used several publicly available datasets: Prostate, DLBCL and Lung cancer. The prostate and DLBCL datasets were both downloaded from http://ico2s.org/datasets/microarray.html, and the lung cancer dataset can be downloaded at http://www.ncbi.nlm.nih.gov/geo with access number [GSE40419].

More information on these datasets is given in Table 2.

**Table 2:** Real datasets used in this paper.

Dataset	No. of Samples (Total)	No. of Genes	Classes
Prostate	102	12600	Normal/Tumor
Lymphoma	77	7129	DLBCL/FL
Lung cancer	164	22401	Normal/Tumor

#### Prostate

This dataset was originally proposed by Singh *et al*. [22]; it is contains the expression profiles of 12,600 genes for 50 normal tissues and 52 prostate tumor tissues.

#### Lymphoma

This dataset (Shipp *et al*. [23]) contains 77 microarray gene expression profiles of the two most prevalent adult lymphoid malignancies: 58 samples of diffuse large B-cell lymphomas (DLBCL) and 19 follicular lymphomas (FL). The original data contains 7,129 gene expression values.

#### Lung cancer

As RNA- sequencing (RNA-seq) technique widely used, therefore, it is important to test the proposed method whether it has the ability to handle the RNA-seq data. To verify it, one dataset that used the next-generation sequencing was involved in our analysis. This dataset [24] contains 164 samples with 87 lung adenocarcinomas and 77 adjacent normal tissues.

**Fig 3:**
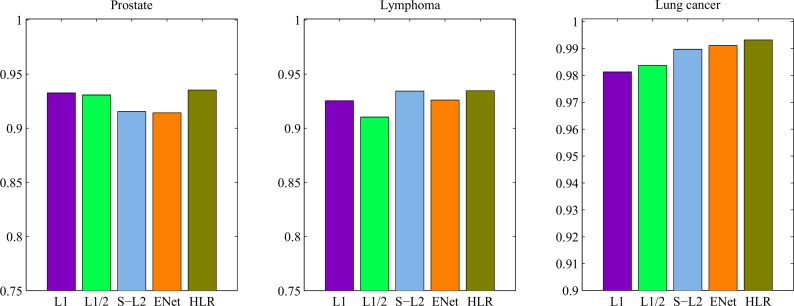
The performance of the AUC from ROC analyzes of each method on prostate, lymphoma and lung cancer datasets.

We evaluate the performance of the HLR penalized logistic regression models using the random partition. This means that we divide the datasets at random such that approximate 75% of the datasets becomes the training samples and the other 25% as the test samples. The optimal tuning parameters were found by using the 10-fold cross-validation in the training set. Then, the classification model was built by the sparse logistic regression with the estimated tuning parameters. Finally, application of the classifier to the test set provides the prediction characteristics such as classification accuracy, AUC under the receiver operating characteristic (ROC) analysis. The above procedures were repeated 500 times with different random dataset partitions. The mean number of the selected genes, the training and the testing classification accuracies, were summarized in Table 3 and the averaged AUC performances were showed in Fig 3.

**Table 3:** Mean results of empirical datasets. In bold–the best performance.

Dataset	Method	Training accuracy (10-CV)	Accuracy (testing)	No. of selected genes
	Lasso	96.22%	92.40%	13.7
	L_1/2_	96.13%	92.18%	8.2
Prostate	SCAD − L_2_	95.99%	91.33%	22
	ElasticNet	96.28%	91.35%	15.2
	HLR	**97.61%**	**93.68%**	12.6
Lymphom					Lasso	96.03%	91.11%	13.2
L_1/2_	95.15%	91.20%	10.7
SCAD − L_2_	95.78%	92.99%	20.9
ElasticNet	96.01%	92.17%	21.2
HLR	**96.55%**	**94.23%**	15.1
Lung cancer					Lasso	96.32%	96.99%	13.8
L_1/2_	97.17%	97.20%	11.5
SCAD − L_2_	97.95%	98.17%	25.1
ElasticNet	97.21%	**98.38%**	28.9
HLR	**98.59%**	98.35%	15.6

*Mean results are based on 500 repeats*.

As showed in Table 3, for prostate dataset, the classifier with the HLR approach gives the average 10-fold CV accuracy of 97.61% and the average test accuracy of 93.68% with about 12.6 genes selected. The classifiers with Lasso, L_1/2_, SCAD − L_2_ and Elastic net methods give the average 10-fold CV accuracy of 96.22%, 96.13%, 95.99%, 96.28% and the average test accuracy of 92.4%, 92.18%, 91.33%, 91.35% with 13.7, 8.2, 22 and 15.2 genes selected respectively. For lymphoma datasets, it can be seen that the HLR method also achieves the best classification performances with the highest accuracy rates in the training and test sets. For lung cancer, our method gained the best training accuracy. The testing performance of Elastic net was slightly better than our method. However, the HLR method achieved its success using only about 15.6 predictors (genes), compared to 28.9 genes for the Elastic net method. Although the Lasso or L_1/2_ methods gained the sparsest solutions, the classification performance of these two approaches were worse than the HLR method. This is an important consideration for screening and diagnostic applications, where the goal is often to develop an accurate test using as few features as possible in order to control cost.

As showed in Fig 3, our proposed method achieved the best classification performances in these three real datasets amongst all the competitors. For example, the AUC from ROC analysis of the HLR method for datasets prostate, lymphoma and lung cancer datasets were estimated to be 0.9353, 0.9347 and 0.9932 respectively. AUC results of the Lasso method for the three datasets were calculated to be 0.9327, 0.9253 and 0.9813 respectively, which were worse than the proposed HLR method.

We summarized the top 10 ranked (most frequently) genes selected by the five regularization methods for the lung cancer gene expression dataset in Table 4, the information of top 10 ranked genes for the other datasets could be found in S2 File. Note that in Table 1, the proposed HLR method has the impressive performances to select the true features in the simulation data. It is implied that the genes selected by the HLR method in these three cancer datasets are valuable to the researchers who want to find out the key factors that associated with the cancer development. For example, in Table 4, the biomarkers selected by our HLR method include advanced glycosylation end product receptor (AGER), which is a member of the immunoglobulin superfamily predominantly expressed in the lung. AGER plays a role in epithelial organization, and decreased express of AGER in lung tumors may conduce to loss of epithelial tissue structure, potentially leading to malignant transformation [25]. The unique function of AGER in lung, making it could be used as an additional diagnostic tool for lung cancer [26], and even a target [27]. GATA2 (GATA binding protein 2) are expressed principally in hematopoietic lineages, and have essential roles in the development of multiple hematopoietic cells, including erythrocytes and megakaryocytes. It is crucial for the proliferation and maintenance of hematopoietic stem cells and multi-potential progenitors [28]. Kumar et al. [29] showed a strong relationship between GATA2 and RAS-pathway mutant lung tumor cells.

**Table 4:** The most frequently selected 10 genes found by the five sparse logistic regression methods from the lung cancer dataset.

Rank	Lasso	L_1/2_	SCAD − L_2_	ElasticNet	HLR
1	STX11	A2M	ABCA8	CCDC69	ACADL
2	GABARAPL1	ACADL	ADH1B	STX11	CCDC69
3	PDLIM2	PNLIP	CAT	GABARAPL1	STX11
4	CAV1	AAAS	CAV1	TNXB	ABCA8
5	ABCA8	A4GALT	CCDC69	PDLIM2	PAEP
6	GPM6A	ABHD8	GABARAPL1	FAM13C	AGER
7	GRK5	ADD2	GPM6A	GPM6A	GATA2
8	TNXB	SLN	GRK5	SFTPC	PNLIP
9	ADH1B	ACTL7B	PDLIM2	ARHGAP44	A2M
10	PTRF	ADAR	PTRF	CAT	ACAN

To further verify the biomarkers selected by our method, we had collected two independent lung cancer datasets for validation. The GSE19804 [30] contains 120 samples with 60 lung adenocarcinomas and 60 adjacent normal tissues. The GSE32863 [31] contains 116 samples include 58 lung adenocarcinomas and 58 healthy controls. These two datasets are available from the GEO series accession number [GSE19804] and [GSE32863].

We used the support vector machine (SVM) approach to build the classifiers based on the first two, first five and first ten genes selected by the different regularization approaches from the lung cancer dataset (Table 4), and were trained on the lung cancer dataset (Table 2) respectively. These classifiers then were applied to the two independent lung cancer datasets, GSE19804 and GSE32863, respectively.

It is known that the obtained prediction models may be only applicable to samples from the same platform, cell type, environmental conditions and experimental procedure. However, interestingly, as demonstrated in Table 5, we can see that all the classification accuracies predicted by the classifiers with the selected genes by the HLR approach, are higher than 90%. Especially the classification accuracy on the GSE32863 dataset is 97.41% with the classifier based on the first ten genes. Such performances are better than the genes selected by other methods. For example, the accuracy of the classifier with the first two genes selected by Elastic net, for GSE19804, was estimated to be 86.67% that was worse than the classifier with the genes selected by our method, 90.83%. The performance of the classifier with the first five genes selected by SCAD − L_2_, for GSE32863, was calculated to be 92.24% that was worse than the classifier with the genes selected by our HLR method, 96.55%. The results indicate that the sparse logistic regression with the HLR approach can select powerful discriminatory genes.

**Table 5:** The validation results of the classifiers based on the top rank selected genes from lung cancer dataset. In bold–the best performance.

Dataset	Method	SVM with the top genes		
		2	5	10
GSE19804					Lasso	89.17%	**93.33%**	92.50%
L_1/2_	85.83%	90.83%	91.67%
SCAD − L_2_	89.17%	89.17%	93.33%
ElasticNet	86.67%	87.50%	89.17%
HLR	**90.83%**	92.50%	**94.17%**
GSE32863					Lasso	93.10%	95.69%	93.97%
L_1/2_	93.97%	94.83%	95.69%
SCAD − L_2_	90.28%	92.24%	94.83%
ElasticNet	89.66%	91.38%	93.97%
HLR	**94.83%**	**96.55%**	**97.41%**

We used the SVM approach to build the classifiers based on the first two, first five and first ten genes selected by the different regularization approaches from the lung cancer dataset (Table 4), and were trained on the lung cancer dataset (Table 2) respectively. These classifiers then were applied to the two independent lung cancer datasets, GSE19804 and GSE32863, respectively.

In addition to comparing with the Lasso, L_1/2_, SCAD − L_2_ and Elastic net techniques, we also make a comparison with the results of other methods for datasets prostate and lymphoma published in the literature. Note that we only considered methods using the CV approach for evaluation, since approaches based on a mere training/test set partition are now widely known as unreliable [32]. Table 6 displays the best classification accuracy of other methods. In Table 6, classification accuracy achieved by the HLR approach is greater than other methods. Meanwhile, the number of selected genes is smaller than other methods except on the Lymphoma dataset.

**Table 6:** The result of the literature. In bold–the best performance.

Dataset	Author	Accuracy (CV)	No. of selected features
	T.K. Paul et al. [33]	96.60%	48.5
	Wessels et al. [34]	93.40%	14
	Shen et al. [35]	94.60%	unknown
prostate	Lecocke et al. [36]	90.10%	unknown
	Dagliyan et al. [37]	94.80%	unknown
	Glaab et al. [38]	94.00%	30
	HLR	**97.61%**	12.6
Lymphoma	Wessels et al. [34]	95.70%	80
	Liu et al. [39]	93.50%	6
	Shipp et al. [23]	92.20%	30
	Goh et al. [40]	91.00%	10
	Lecocke et al. [36]	90.20%	unknown
	Hu et al. [41]	87.01%	unknown
	Dagliyan et al. [37]	92.25%	unknown
	Glaab et al. [38]	95.00%	30
	HLR	**96.55%**	15.1

## 4. Conclusion

In this paper, we have proposed the HLR function, a new shrinkage and selection method. The HLR approach is inherited some valuable characteristics from the L_1/2_ (sparsity) and L_2_ (grouping effect where highly correlated variables are in or out a model together) penalties. We also proposed a novel univariate HLR thresholding function to update the estimated coefficients and developed the coordinate descent algorithm for the HLR penalized logistic regression model.

The empirical results and simulations show the HLR method was highly competitive amongst Lasso, L_1/2_, SCAD − L_2_ and Elastic net in analyzing high dimensional and low sample sizes data (microarray and RNA-seq data). Thus, logistic regression with the HLR approach is the promising tool for feature selection in the classification problem. Source code of sparse logistic regression with the HLR approach was provided in S3 File.

## Supporting Information

S1 File The proof of theorem 1. (PDF)

S2 File The most frequently selected 10 genes information. Top-10 ranked genes selected by all the methods for prostate and lymphoma datasets.
(PDF)

S3 File Source code of the HLR method. MATLAB code of sparse logistic regression with the HLR approach.
(RAR)
