# Supplementary material for: Feature Selection and Cancer Classification via Sparse Logistic Regression with the Hybrid L1/2 +2 Regularization
Source: PLoS One. 2016 May 2;11(5):e0149675. doi: 10.1371/journal.pone.0149675 (PMC4852916; doi:10.1371/journal.pone.0149675)
Supplement: S1 File — (PDF) [file pone.0149675.s001.pdf]

Proof of theorem 1

The proof of theorem 1 is similar to that in Zou and Hastie (2005).

Lemma 1. Given dataset (y, X) and $(\lambda_1,\ \lambda_2)$, define an artificial dataset $(y^*,\ X^*)$ by

$$X^*{}_{(n+p)\times p} = (1+\lambda_2)^{-\frac{1}{2}}\binom{X}{\sqrt{\lambda_2}\boldsymbol{I}},\ Y^*_{(n+p)} = \binom{Y}{0}.$$

Let$\gamma = \lambda_1/\sqrt{1+\lambda_2}$ and $\beta^* = \sqrt{1+\lambda_2}\beta$. Then the HLX criterion can be expressed as

$$L(\lambda_1,\lambda_2,\beta) = L(\gamma,\beta^*) = |y^* - X^*\beta^*|^2 + \gamma|\beta^*|_{1/2}$$

Let

$$\hat{\beta}^* = \operatorname{argmin}_\beta\{L(\gamma,\beta^*)\},$$

Then

$$\hat{\beta} = (1/\sqrt{1+\lambda_2})\ \hat{\beta}^*.$$

Assume $\hat{\beta}$ is the estimates for the HLX regularization. By Lemma 1 and equation (2) we have

$$\hat{\beta} = \operatorname{argmin}_\beta |y^* - X^*\frac{\beta}{\sqrt{1+\lambda_2}}|^2 + \frac{\lambda_1}{\sqrt{1+\lambda_2}}\left|\frac{\beta}{\sqrt{1+\lambda_2}}\right|_{1/2}$$

$$= \operatorname{argmin}_\beta \beta^T\left(\frac{X^{*T}X^*}{1+\lambda_2}\right)\beta - 2\frac{y^{*T}X^*\beta}{\sqrt{1+\lambda_2}} + y^{*T}y^* + \frac{\lambda_1|\beta|_{1/2}}{1+\lambda_2}. \qquad (13)$$

Substituting the identities

$$X^{*T}X^* = \left(\frac{X^TX+\lambda_2}{1+\lambda_2}\right),$$

$$y^{*T}X^* = \left(\frac{y^TX}{\sqrt{1+\lambda_2}}\right),$$

$$y^{*T}y^* = y^Ty$$

into equation (13), we have

$$\hat{\beta} = \operatorname{argmin}_\beta \frac{1}{1+\lambda_2}\left\{\beta^T\left(\frac{X^TX+\lambda_2\mathrm{I}}{1+\lambda_2}\right)\beta - 2y^TX\beta + \lambda_1|\beta|_{1/2}\right\} + y^Ty$$

$$= \operatorname{argmin}_\beta \beta^T\left(\frac{X^TX+\lambda_2\mathrm{I}}{1+\lambda_2}\right)\beta - 2y^TX\beta + \lambda_1|\beta|_{1/2}.$$
