# Supplementary material for: Feature Selection and Cancer Classification via Sparse Logistic Regression with the Hybrid L1/2 +2 Regularization
Source: PLoS One. 2016 May 2;11(5):e0149675. doi: 10.1371/journal.pone.0149675 (PMC4852916; doi:10.1371/journal.pone.0149675)
Supplement: S2 File — Top-10 ranked genes selected by all the methods for prostate and lymphoma datasets. (PDF) [file pone.0149675.s002.pdf]

Table S1. The most frequently selected 10 genes found by the five sparse logistic regression methods from the prostate dataset.

Rank	Lasso	$L_{1/2}$	SCAD-L_2	ElasticNet	HLR
1	PTDSS1	PTGDS	ATP5I	PTDSS1	HPN
2	ATP5I	JUNB	TP63	G0S2	DUSP1
3	S100A4	RP11-124D2.7	PTDSS1	S100A4	NTRK1
4	AGR2	POLR2M	AGR2	TP63	GSTA2
5	XBP1	CYBA	MYOF	XBP1	TP63
6	HPN	RRAD	D4S234E	JUNB	NELL2
7	PTGDS	NR4A3	S100A4	C1QTNF3-AMACR	PTDSS1
8	SDC1	CTSG	PRKCB	FP236383.12	SERINC5
9	JUNB	PRKCB	CTSG	SERPINA3	PHLDA2
10	CFD	ATP5I	ME1	JUND	ATP5I

Table S2. The most frequently selected 10 genes found by the five sparse logistic regression methods from the lymphoma dataset.

Rank	Lasso	$L_{1/2}$	SCAD-L_2	ElasticNet	HLR
1	TLN2	TLN2	ESPL1	GANAB	UPK2
2	ARID4B	IFNA2	UPK2	NUP93	TLN2
3	BRD2	IGLV4-3	IFFO1	MIR4668	CCL21
4	IFFO1	CSTA	PRDX6	UPK2	RRS1
5	RPL24	DLGAP5	IFNA1	OMD	IFFO1
6	PRDX6	CCL21	PNP	TLN2	CIRBP
7	CCL21	IFNA1	IGHEP1	CCL21	PSMA4
8	RP11-419C5.2	BRD2	CCL21	CYP4F3	CCR1
9	UPK2	ADIPOQ	KDR	RPL24	RPL24
10	CIRBP	ESPL1	DTYMK	PRDX6	GANAB
